# Enzootic Bovine Leukosis: Surveillance Measures and Control Program in the Northern Dobruja Area of Romania Between 2017 and 2020

**DOI:** 10.3389/fvets.2021.687287

**Published:** 2021-08-17

**Authors:** Elena Irimia, Madalina Mincu, Elena Narcisa Pogurschi, Jaka Jakob Hodnik, Inge M. G. A. Santman-Berends

**Affiliations:** ^1^Research Department, Research and Development Institute for Bovine Balotesti, Balotesti, Romania; ^2^Department Formative Science in Animal Breeding and Food Industry, University of Agronomic Sciences and Veterinary Medicine of Bucharest, Bucharest, Romania; ^3^Clinic for Reproduction and Large Animals – Section for Ruminants, Veterinary Faculty, University of Ljubljana, Ljubljana, Slovenia; ^4^Department of Population Health Sciences, Faculty of Veterinary Medicine, Utrecht University, Utrecht, Netherlands; ^5^Epidemiology Department, Royal GD, Deventer, Netherlands

**Keywords:** non-regulated disease, enzootic bovine leukosis, dobruja area, surveillance, Romania

## Introduction

Enzootic bovine leukosis (EBL) is a viral disease that affects cattle, characterized by persistent lymphocytosis or lymphosarcoma or both. The epidemiological cause of the EBL is the bovine leukosis virus (BLV). BLV can be transmitted by iatrogenesis, through vertical transmission, via semen, or by hematophagous insects (Order *Diptera*, Family *Muscidae*) (1, 2). The hematophagous insects from the Danube Delta area are vectors for countless diseases (parasitic, viral, and bacterial), being influenced by the favorable wetland environmental conditions (2). Given that the spread of BLV between herds can occur via infected cattle, preventing contact with infected blood is the most important measure for prevention. EBL infection has been associated with direct production losses (decreased milk production, high mortality rates, and reproductive failure) and increased veterinary services costs, leading to reduced export competitiveness (3). Moreover, persistent immunodeficiency and increased susceptibility to other diseases occur in affected animals (4, 5).

EBL represents a major threat for the Romanian cattle sector. Official control measures that are practiced internationally to control EBL include screening or surveillance of cattle for the presence of BLV antibodies to allow intracommunity trade, movement controls within countries, and culling of infected animals. No vaccine is currently available for EBL (6).

The disease was first reported in Romania in 1954, diagnosed in a Dobruja Red bull (7). Eradication efforts have been carried out in Romania since the first detection of the virus, with the EBL laboratory being set up since 1966. The first national regulation for the diagnosis, prevention, and control of EBL was proposed in 1967 (DAS 109 415), and according to the disposition of the act, control and screening for the diagnosis of EBL became mandatory. In 1980, the first diagnostic serological immunodiffusion (agar gel immunodiffusion, AGID) tests were introduced, and EBL was included in the veterinary sanitary law in 1990 (8). Administrative decisions specifying and refining official control measures were made in 2016, when Romania adopted the European Union's legislation for EBL (9) and widespread geographical distribution in Romania is aided by the semi-feral animal husbandry systems practiced in Dobruja, where cows are kept on isolated islands in groups of 20–40 individuals. The diet of cows is reliant exclusively on local unimproved wetland pastures. Especially during summer months, the yield of the pastures is low, and cattle lose body condition. No artificial insemination is practiced; instead, farmers rely on natural bull service, which increases the spread of EBL (10). The cattle population is dominated by crossbreeds between local Romanian Gray cattle and Holstein-Friesian or Aberdeen Angus breeds (11).

The development of effective insect vector control programs (CPs) is not feasible given that the Danube Delta has been a UNESCO World Heritage Site since 1991 (12). Additionally, the area is listed as a *Site of Community Importance* (SCI), as defined in the European Commission Habitats Directive (92/43/EEC).

The overall objective of the current data report was to evaluate EBL surveillance strategies and to highlight the strengths and weaknesses of the strategy that was applied in Romania between 2017 and 2020, in the Danube Delta area.

## Methods

**Figure 1:**
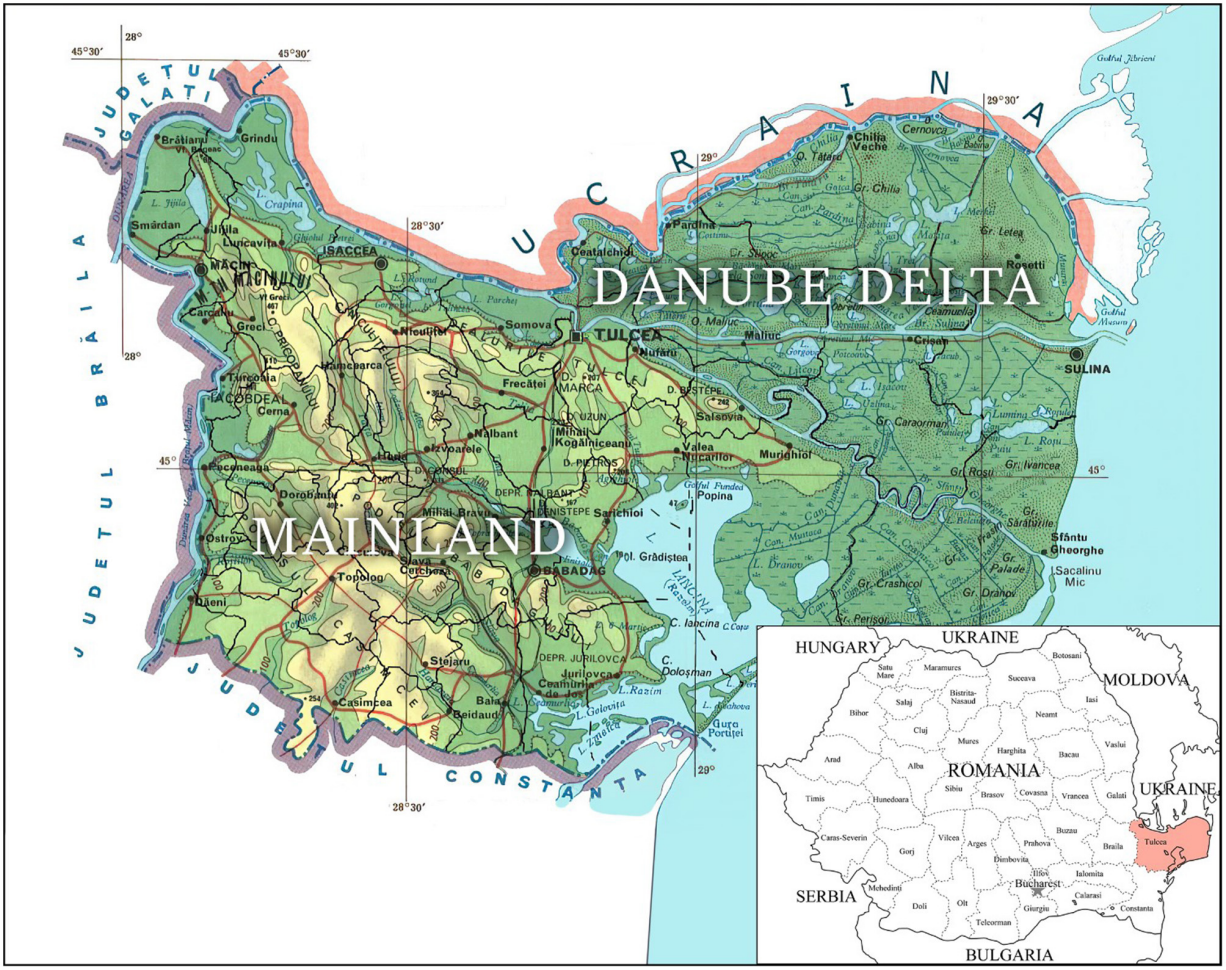
Tulcea County, total area 849.875 ha, with 363.941 ha of agricultural land, 103.545 ha of forests and forest vegetation, 342.132 ha covered with water and ponds, 294.039 ha registered as arable, and 60.597 ha natural pastures^3^^,^^4^.

The study was carried out on EBL records from 2017 to 2020. Official data were obtained from the Veterinary Sanitary Direction of Tulcea County, Romania, and included the whole cattle population in both the mainland and the Danube Delta areas (Figure 1). The EBL control strategy in certain regions of Dobruja is subjected to additional measures, with a tailored local disease CP for eradication and surveillance. The Dobruja area is the southeastern region of Romania and consists of two counties: Constanta and Tulcea; the second one represents the northern part of Dobruja, which in turn includes the mainland and the Danube Delta. A locality is represented by a human settlement forming an administrative unit, village, or town. The map was made by the authors using the Adobe Photoshop CC 2019 program.

## EBL CP

### Legislation and Surveillance Program

Twenty-five European Union countries reported disease CPs in place for EBL (13). Since 2016, in Romania, an official EBL CP has been in place. At enrolment in the CP, holdings in which all animals over 12 months of age have been tested negative for two consecutive tests obtain the official EBL-free status (9). Once a herd is classified as EBL free, all restrictions that are in place for herds without an EBL-free status are withdrawn. In EBL-free herds, serological surveillance in all cattle over 24 months of age is performed once a year, to maintain the official disease-free herd status. Individual blood samples are collected by the local veterinary services and submitted to the laboratory for serological examination by enzyme-linked immunosorbent assay (ELISA). Samples of cattle tested positive by ELISA are confirmed by AGID (9). Seropositive animals have to be slaughtered within 30 days of diagnosis. Furthermore, the farm is placed under official restrictive measures (the herd will be tested and animal movements prohibited) until clinical and serological examinations rule out the presence of EBL-infected cattle. The EBL health status of the herd is suspended until all animals over 12 months of age have reacted negatively to two consecutive serological tests, performed at an interval of at least 4 months and at most 12 months (9). If tumors are detected during meat inspection in slaughtered animals, samples are taken for histopathological examinations. In the case of a histopathological diagnosis that confirms the suspicion of EBL, all animals from the holding of origin are serologically examined.

### Management of Infected Animals

Slaughtering of confirmed EBL-positive cattle is compensated and subsidized up to 100% of market value at the state's expense. Generally, the market value is paid to owners, regardless of whether the animals were slaughtered or died, in order to quickly eliminate outbreaks. The state-subsidized prices depend on sex, age, weight, physiological condition, and production category, according to Government Decision no. 1214/2009. However, testing is not mandatory for animals originating from the Danube Delta, given the semi-feral rearing conditions there. The movement of animals between herds within other counties is allowed after performing a serological test with a negative result, 10 days before the movement (9). The directives for the CP in Romania have evolved to include more details with each new version of the CP at the European Union level, although key features have remained constant. The basic strategy has always involved active surveillance and slaughtering of infected animals and animal transport and movement restrictions within the county.

## Data Validation

Data on the epidemiological situation of EBL were collected from the Veterinary and Food Safety Directorate of Tulcea County, which represents the administrative territorial structure of the national competent authority in the area^1^. For data analysis, descriptive statistics such as incidence and prevalence of EBL with frequency tables were calculated. Parameters such as number of localities with outbreaks, infected animals, and total number of cattle populations for each year were used to calculate the incidence and prevalence of EBL disease per year in Tulcea County. An outbreak in a locality was defined as a spreading point of a disease. The prevalence was calculated as the number of infected animals out of the total number of tested animals. The number of tested animals was based on farmer requests for an EBL evaluation of the cattle in his/her herd. Prevalence rates were calculated with the number of animals for each area separately (Danube Delta and the mainland). Proportion tests (Prtesti) were computed in Stata 15® (StataCorp LLC, USA) and used to compare prevalence and incidence rates between areas and years. Observations with implausible values were removed and were checked for double entries. At the European Union level, there are protocols in force regarding the epidemiological status of each member state and region (14). This work is part of the SOUND-control COST Action based on the epidemiological situation of infectious diseases in cattle and on intra-community economic consequences^2^, aiming to investigate the requirements and necessities for a single general regulatory output-based framework (15).

## Descriptive Results

### Dataset Overview and Description

The outbreaks of EBL occurred in both the mainland area and the Danube Delta. From 2017 until 2020, records of all localities with EBL outbreaks from Tulcea County are presented in Table 1.

**Table 1:** The outbreak situation of EBL in Tulcea County, Romania.

**Year**	**Number of localities**	**Localities with outbreaks**	Outbreak incidence^*^	**Number of animals**	**Infected animals**	**Prevalence at the end of the year**
**Danube Delta**							
2017	36	25	69.4%^a^	24,424	0	0%
2018	36	30	83.3%^a,b^	22,391	265	1.2%
2019	36	19	52.8%^b^	22,513	429	1.9%
2020	36	17	47.2%^b^	23,161	89	0.4%
**Mainland**							
2017	102	41	40.2%^c^	14,978	269	1.8%
2018	102	36	35.3%^c^	16,941	103	0.6%
2019	102	28	27.5%^c,d^	16,765	189	1.1%
2020	102	17	16.7%^d^	15,835	214	1.4%

* *Columns with percentages containing different superscript indicators were significantly different in the proportion test P-value < 0.05*.

In 2018, a non-significant increase of EBL localities with outbreaks was observed in the Danube Delta (*n* = 30), while on the mainland area, the number of infected localities tended to decline, compared to that in 2017. From 2019 onwards, the number of localities with outbreaks decreased in both areas; in the Danube Delta, the total number decreased to 17, while on the mainland, the number of localities with outbreaks decreased to 17. EBL outbreaks were observed on a substantial proportion of localities in Tulcea County (Table 1). Nevertheless, the proportion of localities with outbreaks seemed to decline over time, with significantly fewer outbreaks occurring in 2020 (47.2 and 16.7%, respectively) as compared to 2017 (69.4 and 40.2%, respectively) in both areas of the county. In all studied years, the outbreak incidences at the locality level were significantly higher in the Danube Delta area, compared to the mainland area (proportion test: *P*-value < 0.05). These differences could be attributed to the higher number of cattle raised in the delta region, which facilitates animal-to-animal transmission, or to the more extensive rearing conditions found in the delta, compared to the mainland.

The prevalence at the end of each year varied between 0 and 1.9%. However, because testing is optional,these findings could reflect the level of testing carried out in the area. Moreover, inconsistences between the number of localities and the number of cattle affected by EBL could be attributed to the isolation of outbreaks, combined with the success of the surveillance and eradication program in some of the localities, which have become EBL free. An unexpected pattern was observed in the year 2017 for the number of infected animals in the Danube Delta. This might be attributed to the transcription of the outbreaks from 1 year registered in the following year; e.g., no farmer requested animal testing for EBL during that year.

During the current study, only 15 farms in the Danube Delta area were classified as free of EBL, with 13 farms in 2018 and a further two in 2019. These 15 farms were the only ones that fulfilled the eligibility conditions for the EBL-free status, with a negative response to the serological tests. For the remaining herds, eligibility for free status was impossible, due to animals that tested positive found within the remaining tested herds.

## Discussions

The differences for EBL incidences between the Danube Delta and the mainland of Tulcea County could be the result of existing legislation, which states that all mainland cattle owners are required to test their animals on an annual basis, as part of the county-level surveillance program. However, cattle owners from the Danube Delta do not have to comply with these regulations (different testing strategies), resulting in a likely underestimation of the EBL incidence in the region. Prevalence observed in this data report is an overestimation of the animal-level prevalence in the whole country (9). EBL outbreaks were also reported in other Southeastern European regions, including neighboring countries, such as Ukraine and Bulgaria (16). It is believed that horizontal transmission through insect vectors is one of the major transmission routes. Both the EBL transmission paths and the level of precipitation and temperature in the Tulcea area could have influenced the extent of disease transmission. The only local cattle breed, Dobruja Red, is officially regarded as extinct due to the high susceptibility of the breed to EBL (10, 11). Conversely, in a recent study conducted in southwestern Romania in 2017, where 27,701 adult cattle were tested, no outbreaks or positive serological cases were found (12). Although previous studies on the genetic merit of sires for leukosis resistance provided low heritability estimates for Holstein (0.08) and Jersey (0.07) cattle breeds (17), the authors recommended that this could still provide value in controlling the infection. Considering management procedures, several studies suggested that instead of testing and culling seropositive animals, it is more cost-effective to obviate disease transmission by implementing preventive practices, e.g., movement restrictions and vector controls (18).

A high average annual temperature (+10°C) and being the region with the least precipitation in Romania (300–450 mm/year) (19) favor EBL transmission. Although the cattle density in Tulcea County is low, with only 0.02 breeding cattle/100 ha, compared to 0.13 breeding cattle/100 ha at the national level, it remains the area with the highest incidence of EBL.

The current CP for EBL in the Danube Delta could be significantly improved if data collection and disease event reporting in small size farms (more than 90% of all reared cattle) were refined (20). Furthermore, the low numbers of state veterinarians in Tulcea County has led to low rates of diagnosis and limited control of outbreaks (21).

The delta region within Tulcea County is problematic, when it comes to EBL spread and prevention measures, given the environmental protected status of this UNESCO site, adding to the semi-feral extensive rearing conditions of bovines. Thus, insect management and extermination policies cannot be applied, while the wetland climate sustains a high and diverse number of vectors (12). Due to the extensive rearing conditions of cattle from this area, there is a lack of official data on the exact number of herds. Based on the Romanian national legislation, the minimum number for a cattle farm to be recognized is five heads; however, there are many semi-subsistence cattle owners who have one to two heads, and all animals graze in communal pastures for several months/year; as a result, there is a lack of official data on this matter.

This paper is the first description of the EBL surveillance and infection status in these areas of Romania. We believe that a European EBL Center for Diagnosis and Control would help significantly to design policies and measures aimed at eradicating the disease at the continental level, while know-how and successful CP stories could be adopted in countries facing EBL outbreaks worldwide.

## Conclusions

This data report shows continuing improvement in the Romanian epidemiological situation concerning EBL. To accomplish the objective of total EBL eradication, effective collaboration between veterinary services, farmers, and local administrative institutions is required.

## Data Availability Statement

The data analyzed in this study is subject to the following licenses/restrictions: The dataset presented in this report is not readily available, being confidential data. Requests to access the dataset should be directed to the regional division of the National Veterinary and Animal Safety Authority, Veterinary and Food Safety Directorate Tulcea. Requests to access these datasets should be directed to Veterinary and Food Safety Directorate Tulcea, http://tulcea.dsvsa.ro/.

## Ethics Statement

The animal study was reviewed and approved by Scientific and ethics committee of the Research and Development Institute for Bovine Balotesti.

## Author Contributions

All authors listed have made a substantial, direct and intellectual contribution to the work, and approved it for publication.

## Conflict of Interest

The authors declare that the research was conducted in the absence of any commercial or financial relationships that could be construed as a potential conflict of interest.

## Publisher's Note

All claims expressed in this article are solely those of the authors and do not necessarily represent those of their affiliated organizations, or those of the publisher, the editors and the reviewers. Any product that may be evaluated in this article, or claim that may be made by its manufacturer, is not guaranteed or endorsed by the publisher.

## Footnotes

^1^http://tulcea.dsvsa.ro/.

^2^https://sound-control.eu/.

^3^Figure: https://upload.wikimedia.org/wikipedia/commons/9/9d/Harta-judetului-Tulcea.jpg.

^4^Area: Tulcea County Council: https://www.cjtulcea.ro/sites/cjtulcea/PrezentareaJudetului/Pages/Economie.aspx.
